# Positive expression of NR6A1/CT150 as a predictor of biochemical recurrence-free survival in prostate cancer patients

**DOI:** 10.18632/oncotarget.11749

**Published:** 2016-08-31

**Authors:** Gong Cheng, Shangqian Wang, Xiao Li, Shuang Li, Yang Zheng, Lei Zhang, Meiling Bao, Chao Liang, Zhengkai Huang, Yiyang Liu, Chao Qin, Pengfei Shao, Jie Li, Lixin Hua, Changjun Yin, Zengjun Wang

**Affiliations:** ^1^ Department of Urology, the First Affiliated Hospital of Nanjing Medical University, Nanjing, China; ^2^ Department of Pathology, the First Affiliated Hospital of Nanjing Medical University, Nanjing, China; ^3^ Department of Urology, the Affiliated Cancer Hospital of Jiangsu Province of Nanjing Medical University, Nanjing, China

**Keywords:** NR6A1, CT antigens, prostate cancer, gene silencing, EMT

## Abstract

NR6A1/CT150, as an orphan receptor, is a novel member of the cancer-testis (CT) antigen family. Here, we investigated the expression and function of NR6A1 and its underlying mechanisms in prostate cancer (PCa) patients who underwent radical prostatectomy. A total of 303 cases of prostate cancer after radical prostatectomy were analysed in a tissue microarray (TMA) for NR6A1 immunohistochemistry-based protein expression. Kaplan–Meier/log-rank analysis and Cox regression analysis were used to investigate the relationship between NR6A1 expression and clinicopathological factors in PCa. NR6A1 mRNA expression was examined by reversing transcriptase-polymerase chain reaction (RT-PCR). Knockdown of NR6A1 by small interfering RNA mediated gene silencing and overexpression of NR6A1 through lentivirus were utilized to investigate its potential role in prostate cancer cells. NR6A1 protein expression was 29.7% (90/303) and mRNA expression was 28.1%(9/32) in PCa patients. NR6A1 expression was significantly associated with Gleason score (GS) (P=0.003) and tumor stage (P=0.042). The patients with positive NR6A1 expression have a shorter biochemical recurrence-free survival. NR6A1 predicted biochemical recurrence in univariate (P=0.0159) and multivariate models (P=0.0317). In addition, gene silencing of NR6A1 resulted in G0/G1 phase cell cycle arrest, and decreased metastatic and invasive potential of prostate cancer cells DU145 and PC3. In contrast, overexpression of NR6A1 reduced G0/G1 phase cell cycle arrest, and promoted metastatic and invasive potential of prostate cancer cells 22RV1. And overexpression of NR6A1 significantly promoted tumor growth *in vivo*. What's more, down regulation of NR6A1 could reverse epithelial-to-mesenchymal transition (EMT) process in DU145 and PC3 cell lines, and the overexpression could enhance EMT process in 22RV1 cell line. NR6A1 played a prominent role in migration and invasion of PCa cells, and it is indicated that NR6A1 may act as a novel marker for biochemical recurrence after radical prostatectomy.

## INTRODUCTION

Prostate cancer (PCa) is one of the most commonly diagnosed cancers worldwide in men and the second cause of cancer-associated mortality in American men [1]. Recently, the incidence and mortality of PCa have been increasing in China, especially in elderly men [2]. The introduction of serum prostate-specific antigen (PSA) testing in the 1980s is thought to have remarkably improved the diagnosis and prognosis of PCa in men. However, PSA is not cancer-specific, and as a biomarker, PSA has some limitations for early diagnosis of PCa, which may result in the over detection and over treatment of this indolent disease [3]. Although major efforts have been made to search for new biomarkers, clinical management of prostate cancer still faces challenges.

**Figure 1:**
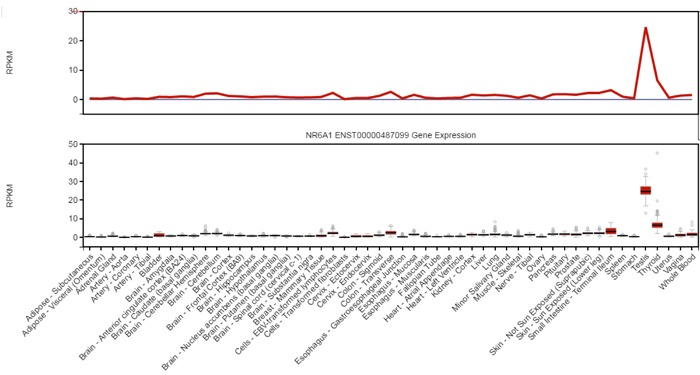
RNA-seq data from human multiple organs demonstrates the testis specific expression of NR6A1 From GTEx database (V6 dbGaP Accession phs000424.v6.p1).

Cancer/testis antigens (CTAs) are a group of tumor-associated antigens that are typically restricted to adult testis, but they are aberrantly expressed in several types of cancers, especially in advanced cancers with stem-like characteristics [4]. Nuclear receptor subfamily 6, group A, member 1(NR6A1/CT150), is an orphan member of the nuclear receptor superfamily that has a common modular structure and shares several functional domains involving DNA binding, nuclear localization, dimerization, and transactivation [5, 6]. We also did the bioinformatic analysis by comparing the expression level of NR6A1 in multiple organs with RNA-seq data. The results showed testis specific expression (Figure 1). As an orphan receptor, NR6A1 is also named germ cell nuclear factor (GCNF) or retinoic acid receptor-related testis-associated receptor (RTR) [7, 8]. NR6A1 is located at chromosome 9q33.3[9]. NR6A1 may act as a transcriptional repressor that plays an important role in mouse embryonic development [10]. It is differentially regulated during retinoid-induced differentiation of embryonal carcinoma and embryonic stem cells [11, 12]. Few articles have reported NR6A1 expression in carcinoma in the past years. However, recently, it has been reported that NR6A1 is lower expressed in ER+ and ER- breast cancer compared with normal mammary gland tissue, and low levels of NR6A1 in cancer cells exhibit higher sensitivity to the anticancer drug ecteinascidin[13]. In addition, NR6A1 levels may be correlated with tumor characteristics in prostate cancer [14]. NR6A1 protein was found higher in prostate cancer patients compared with those with normal prostate tissue. Furthermore, increased NR6A1 immunoreactivity was significantly associated with disease progression in PCa [14]. This gene may act as a promising biomarker of PCa aggressiveness.

Epithelial-to-mesenchymal transition (EMT) is a transdifferentiation process in which adherent epithelial cells convert to motile mesenchymal cells, contributing to prostate cancer progression [15, 16]. The loss of E-cadherin expression has been associated with the progression and metastasis of several malignancies including PCa [17, 18]. Reduced E-cadherin expression is associated with various indices of prostate cancer progression, such as grade, local invasiveness, dissemination into the blood, and tumor relapse after radiotherapy [19]. Also, E-cadherin loss could enhance proliferation and stemness in PCa cells [20]. In contrast, the increased expression of N-cadherin has been reported in various cancers, and is associated with tumor progression and metastasis. For example, the expression of N-cadherin is up-regulated and induces cell migration in prostate cancer [21, 22]. Therefore, silencing N-cadherin abolishes prostate cancer cells migration in three-dimensional (3D) matrix. Another study showed that ZEB-1 acted as a metastatic PCa biomarker [23]. All these studies highlight the relationship between EMT and prostate cancer progression.

In our study, we aimed to determine the expression and function of NR6A1 in PCa tissues and the possible mechanism. Then, we utilized siRNA and lentivirus respectively to make knockdown and overexpression of NR6A1 in PCa cells and investigated the migrative, invasive potentials of PCa. And we also found NR6A1 expression could affect the abnormal expression of E-cadherin, N-cadherin, Vimentin and ZEB-1 in PCa cells. All these results suggested that NR6A1 may be a novel biomarker and this promises a therapeutic strategy for the treatment of PCa.

## RESULTS

### Relationship between NR6A1 expression and clinicopathological factors in PCa

To analyze the function of NR6A1 in PCa, we determined NR6A1 protein expression in 303 cases of prostate cancer. Immunostaining showed that the percentage of NR6A1 positivity was 29.7% (90/303) in PCa samples. The relationship of NR6A1 expression and clinicopathologic characteristics of patients is listed in Table 2. There was no significant difference between NR6A1 expression and age or preoperative PSA (P>0.05). However, NR6A1 expression was significantly associated with Gleason score (GS) (P=0.003), Tumor stage (P=0.042) and Biochemical recurrence (P=0.010). The clinicopathological results indicate that increased NR6A1 expression is associated with advanced prostate cancer.

**Table 1:** Characteristics of the 303 patients treated by radical prostatectomy

Age		
Mean±SD(year)	69.1±6.5	
<60	33	10.9%
60-70	126	41.6%
>70	144	47.5%
Pre-operative PSA(ng/ml)		
<10	74	24.4%
10-20	80	26.4%
>20	149	49.2%
Gleason score		
<7	64	21.1%
≥7	239	78.8%
Pathological stage		
pT2	188	62.0%
pT3/ T4	115	38.0%
Time to PSA progression		
Mean ± SD (months)	16.3±14.8	
Overall follow-up		
Mean ± SD (months)	26.1±17.3	

SD, standard deviation

**Table 2:** Relationship of NR6A1 expression and clinicopathologic characteristics of patients

Variable	NR6A1 expression		
	Negative (n=213)	Positive (n=90)	*P* value
Age			0.821
<60	23	10	
60-70	91	35	
>70	99	45	
Preoperative PSA(ng/ml)			0.119
<10	52	22	
10-20	63	17	
>20	98	51	
Gleason score			**0.003**
<7	48	16	
≥7	165	74	
T stage			**0.042**
pT2	140	48	
pT3/ T4	73	44	
Biochemical recurrence			**0.010**
negative	151	50	
positive	62	40	

Bold: *P* < 0.05. *P* values were two-tailed and based on the Pearson chi-square test.

### NR6A1 expression in PCa tissues and prostate cancer cell lines

NR6A1 mRNA expression was analyzed in 32 PCa samples by RT-PCR (Figure S2). The patients information are summarized in Table S1. Our results indicated that NR6A1mRNA was only expressed in 9 of the 32 (28.13%) PCa tissues. In addition, we selected five cell lines, WPMY-1, LNCaP, DU145, PC3, and 22RV1, to investigate their NR6A1 expression levels as determined by Western blot. High expression of NR6A1 was observed in prostate cancer cell lines (DU145, PC3, LNCaP, and 22RV1), whereas WPMY-1 showed lower expression of NR6A1 (Figure S3). So, DU145 and PC3 were chosen for subsequent knockdown experiments. Moreover, because of the relatively lower expression of NR6A1 in 22RV1, this cell line was selected for overexpression experiments.

### Knockdown of NR6A1 in DU145 and PC3 and cell function assay

**Figure 2:**
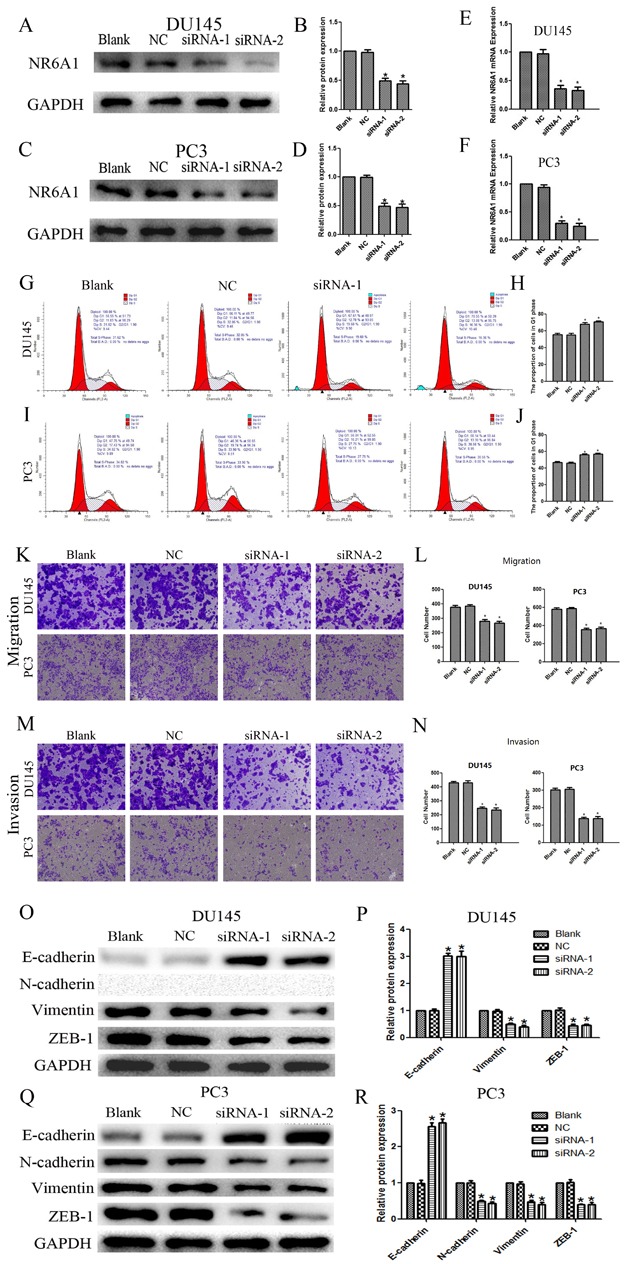
(A)(B) NR6A1 protein expression after 48 hours of transfection with NR6A1 siRNA or NC in DU145 (C)(D) NR6A1 protein expression after 48 hours of transfection with NR6A1 siRNA or NC in PC3. GAPDH was used as a loading control. (E) NR6A1 mRNA expression in DU145 after 24 hours of transfection with NR6A1 siRNA or NC. (F) NR6A1 mRNA expression in PC3 after 24 hours of transfection with NR6A1 siRNA or NC. (G)(H) The knockdown of NR6A1 resulted in G0/G1 phase cell cycle arrest in DU145. (I)(J) The knockdown of NR6A1 resulted in G0/G1 phase cell cycle arrest in PC3. Cell-cycle analysis was performed at 48 hours post-transfection by staining DNA with propidium iodide prior to flow cytometry. Cells transfected with NR6A1 siRNA arrest in G1 phase are compared with Blank and NC. (K)(L) Decreased NR6A1 levels inhibited migration in DU145 and PC3. (M)(N) Decreased NR6A1 levels inhibited invasion in DU145 and PC3. Original magnification 100×. (O)(P) Western blot analysis was used to detect the changes in EMT markers in DU145. Down-regulated expression of NR6A1 reversed changes in EMT markers’ expression with a gain in E-cadherin expression and a loss of vimentin and ZEB-1 in DU145. N-cadherin was not expressed in DU145. (Q)(R) Western blot analysis was used to detect the changes in EMT markers in PC3. Down-regulated expression of NR6A1 reversed changes in EMT markers’ expression with a gain in E-cadherin expression and a loss of N-cadherin, vimentin and ZEB-1 in PC3. GAPDH was used as a loading control. All data are presented as mean± SD of at least three independent experiments. *P < 0.05 compared with Blank or NC.

To investigate the effects of NR6A1 on cell cycle progression in DU145 and PC3 cells, negative control siRNA and NR6A1 siRNA-transfected cells were cultured for 48h. Decreased NR6A1 levels were confirmed by Western blot analyses (Figure 2A, and 2C, 2D) and real-time qRT-PCR (Figure 2E and 2F). The percentages of cells in the G0/G1, S and G2/M phases were determined by using flow cytometry. Cell cycle analyses showed that NR6A1 siRNA transfection increased the percentage of DU145 and PC3 cells in the G0/G1 phase. These data suggested that the knockdown of NR6A1 induced cell cycle arrest at the G0/G1 phase in DU145 (Figure 2G and 2H) and PC3 cells (Figure 2I and 2J) (all P<0.05).

To further confirm the effects of NR6A1 on the metastasis of prostate cancer cells, DU145 and PC3 were transfected with NR6A1 siRNA or NC. The migratory and invasive properties of cells were determined by transwell chamber assay. The results showed that cells with lower expression of NR6A1 displayed significantly decreased migration (Figure 2K and 2L) and invasion (Figure 2M and 2N) compared with NC and Blank (all P<0.05).

As previously described, EMT is a key process in cancer metastasis. Based on the association between NR6A1 expression and the migration and invasion of cancers, we compared the expression of epithelial and mesenchymal markers in cancer cells using Western blotting. Down-regulated expression of NR6A1 reversed changes in EMT markers’ expression with a gain in E-cadherin expression and a loss of vimentin and ZEB-1 in DU145 (Figure 2O and 2P) and PC3 (Figure 2Q and 2R). Whereas, N-cadherin was expressed in PC3, but not in DU145. And this result conformed to the study of Nalla AK [27].

### Overexpression of NR6A1 in 22RV1 and cell function assay

**Figure 3:**
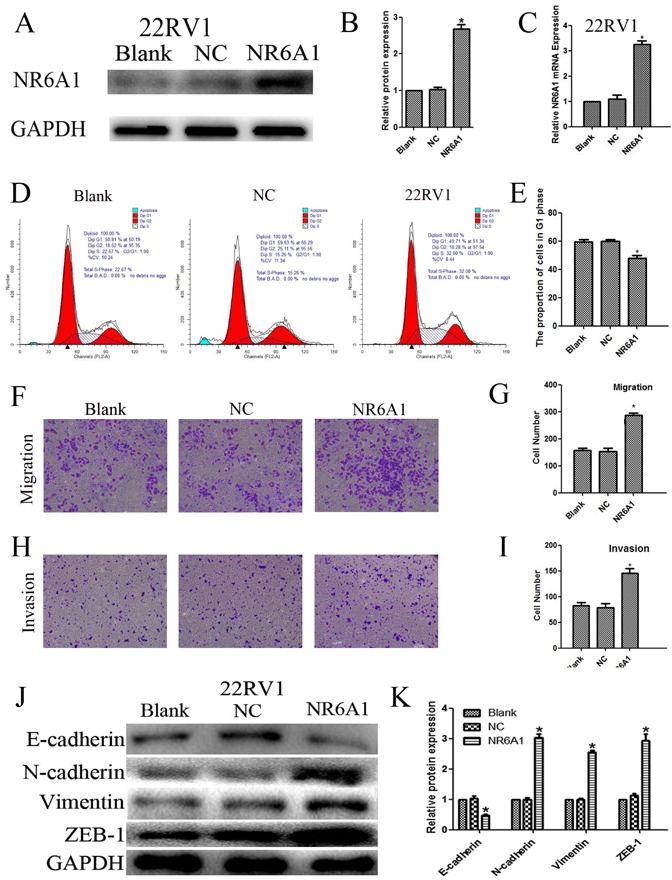
(A)(B) NR6A1 protein expression of transfection with NR6A1 lentivirus or NC in 22RV1 GAPDH was used as a loading control. (C) NR6A1 mRNA expression in 22RV1 after stable transfection with lentivirus or NC. (D)(E) The overexpression of NR6A1 prevented G0/G1 phase cell cycle arrest in 22RV1. (F)(G) Increased NR6A1 levels promoted migration in 22RV1. (H)(I) Increased NR6A1 levels promoted invasion in 22RV1. Original magnification 100×. (J)(K) Western blot analysis was used to detect the changes in EMT markers in 22RV1. Up-regulated expression of NR6A1 promoted changes in EMT markers’ expression with a loss in E-cadherin expression and a gain of vimentin and ZEB-1 in 22RV1. GAPDH was used as a loading control. All data are presented as mean± SD of at least three independent experiments. *P < 0.05 compared with Blank or NC.

In contrast, lentivirus was utilized to induce overexpression of NR6A1 explore the effects on cell cycle progression. Enhanced NR6A1 levels were also confirmed by western blot analyses (Figure 3AB) and real-time qRT-PCR (Figure 3C). Cell cycle analyses indicated that NR6A1 overexpression decreased the percentage of 22RV1 cells in the G0/G1 phase, showing that the overexpression of NR6A1 reduced cell cycle arrest at the G0/G1 phase in 22RV1 (Figure 3D and 3E) (P<0.05).

Also, 22RV1 was transfected with lentivirus to increase NR6A1 expression, in order to study the effects on the metastasis. The results indicated that cells with higher expression of NR6A1 revealed obviously enhanced migration (Figure 3F and 3G) and invasion (Figure 3H and 3I) (all P<0.05). So, NR6A1 may play an important role in PCa progression.

On the contrary, up-regulated expression of NR6A1promoted changes in EMT markers’ expression, including a loss in E-cadherin expression and a gain of vimentin and ZEB-1 in 22RV1 (Figure 3J and 3K).

### Overexpression of NR6A1 significantly promoted cellular growth *in vivo*

**Figure 4:**
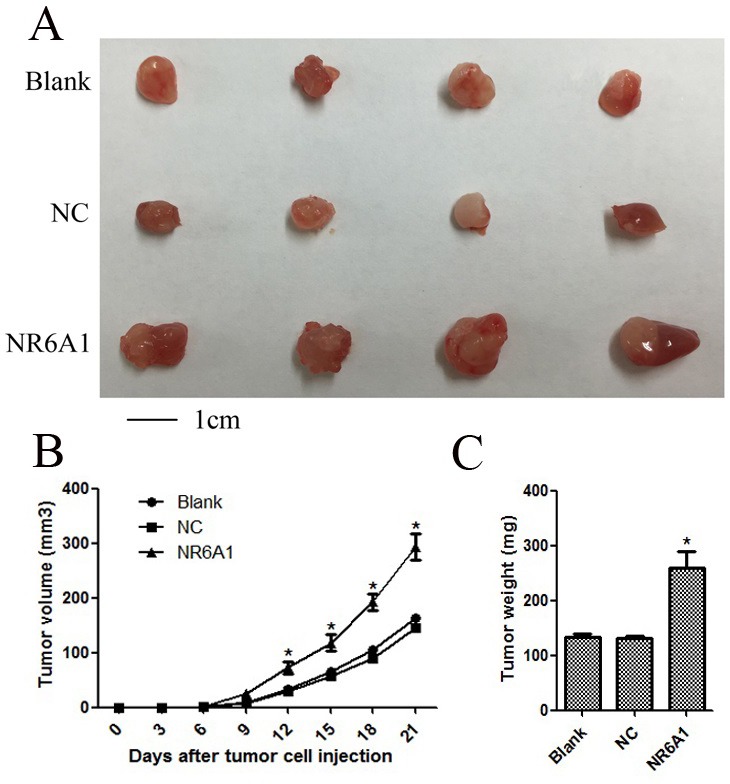
Overexpression of NR6A1 significantly inhibited cellular growth *in vivo* (A) Representative pictures of tumors. (B) Tumor volumes were measured at the indicated number of days after mice were injected with tumor cells. (C) Final weight of tumors from each lentivirus treatment group comparing NC and Blank groups was shown. Each bar represented the mean tumor volume ± S.D. or tumor weight ± S.D. of four mice per group. *P < 0.05 compared with Blank or NC.

To investigate the effects of NR6A1 expression on prostate cancer cell growth *in vivo*, 22RV1 was transfected with lentivirus and then xenografted into nude mice associated with NC and Blank. As shown in Figure 4, tumors derived from 22RV1 cells with overexpression of NR6A1 grew much faster than those derived from 22RV1 Blank and NC cells, which were consistent with *in vitro* results. The results demonstrated that overexpression of NR6A1 significantly promoted tumor growth.

### NR6A1 expression and biochemical recurrence-free survival

**Figure 5:**
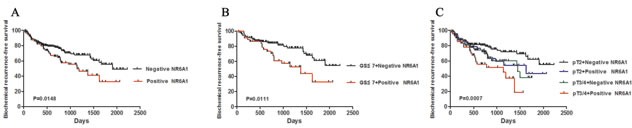
NR6A1 expression and biochemical recurrence (A) Positive NR6A1 expression was associated with reduced biochemical recurrence-free survival. (B) Univariate Kaplan -Meier/log-rank analysis of biochemical recurrence-free survival versus NR6A1 expression and GS≤7 cases. (C) Univariate Kaplan -Meier/log-rank analysis of biochemical recurrence-free survival versus NR6A1 expression vs tumor stage.

We further examined the impact of NR6A1 expression on the clinical outcome of prostate cancer patients. Univariate Cox regression analysis showed that GS≥7 (HR, 0.319, CI=0.166-0.614,P=0.0006), Tumor state (pT3/4 tumors) (HR, 0.580, CI=0.391-0.861,P=0.0068), NR6A1 expression (HR, 0.612, CI=0.411-0.912,P=0.0159) and preoperative PSA(HR, 1.015, CI=1.007-1.023, P=0.0004) were all associated with a significantly shorter biochemical recurrence-free survival (Table 3). In addition, univariate Kaplan–Meier/log-rank analysis also indicated that positive NR6A1 protein expression was significantly related to an increased risk for poor clinical outcomes in PCa patients (log rank P=0.0148; Figure 5A). Patients with positive NR6A1 expression had a shorter disease-free survival.

**Table 3:** Univariate and multivariate analyses of factors associated with biochemical recurrence

Variable	Hazard	95%	
	ratio	CI	*P* value
Univariate analysis			
Age	0.984	0.954-1.015	0.3126
Gleason score(<7 vs ≥7)	0.319	0.166-0.614	0.0006
T stage(pT2 vs pT3/4)	0.580	0.391-0.861	0.0068
NR6A1(negative vs positive)	0.612	0.411-0.912	0.0159
Preoperative PSA	1.015	1.007-1.023	0.0004
Multivariate analysis			
Age	0.987	0.956-1.018	0.4070
Gleason score(<7 vs ≥7)	0.378	0.910-0.752	0.0055
T stage(pT2 vs pT3/4)	0.903	0.586-1.389	0.6410
NR6A1(negative vs positive)	0.643	0.430-0.962	0.0317
Preoperative PSA	1.011	1.001-1.020	0.0199

Multivariate analyses, Cox proportional hazards regression mode. CI, confidence interval.

Furthermore, the Cox multiple variable analysis revealed that GS≥7 (HR, 0.378, CI=0.910-0.752, P=0.0055), positive NR6A1 expression (HR, 0.643, CI=0.430-0.962,P=0.0317) and preoperative PSA (HR, 1.011, CI=1.001-1.020,P=0.0199) were significant prognostic factors of biochemical recurrence (Table 3). The data showed that the NR6A1 expression was a powerful predictor of biochemical recurrence after radical prostatectomy. To analyze a homogeneous group of patients, combining NR6A1 expression and pathological stage was used to stratify the possibility of biochemical recurrence. The positive NR6A1 staining had a significantly increased possibility of biochemical recurrence among the subsets of GS≤7 tumors and pT3/4 compared with those with NR6A1 negative expression(Figure 5B, log rank P=0.0111; Figure 5C, log rank P=0.0007). These results suggest that positive NR6A1 expression may act as a significant prognostic indicator for PCa.

## DISCUSSION

Recently, for patients with PCa, efficient prognostic biomarkers are limited except for PSA. Thus, it is necessary to evaluate some new prognostic biomarkers for patients with this disease. The aim of this study is to indicate that NR6A1 is a positive regulator of prostate cancer progression. And our results demonstrated that NR6A1 expression could be an important prognostic indicator for biochemical recurrence of patients with PCa after radical prostatectomy.

NR6A1 has been shown to be expressed in maturing male germ cells in mouse models [8, 28, 29], and plays a vital role during embryogenesis and gametogenesis in the mouse [10, 30, 31]. Additionally, this gene is frequently expressed in the normal mammary gland tissue, and is lower expressed in ER+ and ER- breast cancer [13]. Here, we analyzed the expression of NR6A1 on samples obtained from 303 PCa samples. The results revealed that NR6A1 protein was expressed in 29.7% of total PCa patients, and the percentage of NR6A1 mRNA expression was 28.13% (9/32). Furthermore, positive NR6A1 expression was found to be associated with GS and tumor stage in PCa. After performing univariate and multivariate analyses, NR6A1 positive expression was found as a significant predictor for biochemical recurrence of PCa patients. More importantly, we found that the patients who had positive NR6A1 expression together with having tumor stage pT3/4 increased risk of biochemical recurrence compared with the other groups. Using the similar statistical analysis, the results showed that in subset of low-middle grade (GS≤7), patients with positive NR6A1 expression had a shorter biochemical recurrence-free survival than GS≤7 and negative NR6A1 expression. Our results strongly suggest that NR6A1 could be of clinical value as a prognostic factor for biochemical recurrence.

Our study revealed that NR6A1 was preferentially expressed in PCa cells of DU145, PC3 and 22RV1, and was nearly negative in the normal prostate stromal cell of WPMY-1. Furthermore, gene silencing approach was employed in this study to investigate this gene and its potential biological effects on tumor progression and metastasis. And, we found that NR6A1 expression was associated with the cell cycle, the migration and invasion of prostate cancer cells. Our data showed that NR6A1 knockdown significantly reduced the ability of migration and invasion of PCa cells, while NR6A1 overexpression obviously increased the ability. We believed that this gene may act as a predictor of tumor progression and metastasis for PCa patients.

Our results clarified the potential functional role of NR6A1 in PCa progression. In addition, our data also suggested that the siRNA-mediated down-regulation of NR6A1 expression in prostate cancer cells of DU145 and PC3 could reverse the epithelial phenotype and repress a mesenchymal phenotype, and overexpression of NR6A1 in 22RV1 could promote EMT progression. EMT is a complicated multistep process, which has been shown to induce epithelial to mesenchymal transition and endow transformed epithelial cells with stem-cell like properties. Recent evidences note that EMT plays an important role in neuroendocrine differentiation, chemoresistance, disease aggressiveness and poor prognosis of prostate cancer [32–34]. Moreover, NR6A1 has been shown to change cell-fate, including cell growth and differentiation, neurogenesis and germ cell differentiation [7, 8, 35–37]. And Mathieu et al speculated that NR6A1 might contribute the molecular pathways leading to neuroendocrine differentiation in PCa [14]. Even this theory has not yet been proven till now, there might still be a strong association between NR6A1 and EMT. Interestingly, our findings showed that down regulation of NR6A1 protein expression significantly increased E-cadherin expression, and markedly reduced N-cadherin, Vimentin and ZEB-1 expression in prostate cancer cells (DU145 and PC3), while overexpression of NR6A1 hold the opposite effects. Therefore, our study suggested that this gene may be a new biomarker for aggressiveness of PCa. However, further questions require to be solved about whether NR6A1 directly takes part in EMT, or whether there exists another target of NR6A1 which may induce EMT.

In summary, our results showed that positive NR6A1 expression was an important independent maker for biochemical recurrence of PCa patients. And silencing expression of NR6A1 in prostate cancer cells by specific siRNA induced cell cycle arrest at the G0/G1 phase, and significantly decreased their invasive and metastatic potentials. While overexpression of NR6A1 hold the opposite function. NR6A1 could play a crucial role in PCa progression, including migration and invasion. However, further studies will be required to verify the mechanism by which NA6A1 induces EMT, and to ensure the roles of NR6A1 *in vivo*.

## MATERIALS AND METHODS

### Patients and Tissue Micro Arrays (TMAs)

The PCa patients for the creation of TMAs in this study have been described previously in detail [24]. Briefly, 303 prostate cancer tissues were obtained from patients who were treated by radical prostatectomy between 2008 and 2013 at the First Affiliated Hospital of Nanjing Medical University (Nanjing, China). All patients were recruited following informed consent. In the present study, the clinical and pathologic features of all patients were summarized in Table 1. Biochemical recurrence (BCR) was defined as two consecutive increases postoperative PSA 0.2 ng/ml or greater in serum. The follow-up deadline was December 2014. The protocols used in the study were approved by the ethics committee of the hospital. Tissue microarrays were constructed with PCa tissues obtained from the above mentioned samples, including 4 cores (0.6 mm diameter) per cancer. This study was approved by the medical ethics committee of the hospital.

### Immunohistochemistry

Serial sections from TMA blocks were deparaffinized in xylene and rehydrated through an ethanol gradient, then were blocked in hydrogen peroxide in methanol for 10 min. Antigen retrieval was performed by incubation for 2 min in a steam pressure cooker containing citrate buffer 10 ml, pH 6.0. Then samples were blocked for 5 min and incubated overnight with antibodies against NR6A1(1:100) at 4°C overnight. After having been washed by phosphate buffer saline (PBS) for 10 min, slides were cultured in the secondary antibody for 30 min. After a 10 min wash in PBS, the antibody reaction was visualized with a fresh substrate solution containing DAB (3,3′-diaminobenzidine). The sections were counterstained with hematoxylin, dehydrated, and coverslipped.

### Evaluation of staining

Evaluation of protein staining was separately and independently performed by two experienced pathologists without knowledge of the clinical data. The results of immunohistochemical staining for NR6A1 were determined by the Amend Allred scoring system as described in a previous study [25, 26]. Briefly, the percentage of positive tumor cells was determined in at least five areas at 400×magnification and assigned to one of the following five categories: 0,<5%; 1, 5–25%; 2, 25–50%; 3, 50–75% and 4,>75%. The intensity of immunostaining was scored as follows: 1, low; 2, moderate and 3, strong. Given the homogenicity of the staining of the target proteins, the predominant pattern was taken into account for scoring. The immunohistochemical scores of PCa tissues for NR6A1 were: negative expression (<1) and positive expression (1-12).

### Specimens and cell culture

A total of 32 PCa tissues were included in this study. Tissues were obtained from patients who underwent radical prostatectomy at Department of Urology of the First Affiliated Hospital of Nanjing Medical University (Nanjing, China). The specimens were snap frozen in liquid nitrogen after surgery and stored at −80°C until use. The human PCa cell lines (DU145, PC3, LNCaP, and 22RV1), normal prostate stromal cell (WPMY-1) were purchased from the Cell Bank Type Culture Collection of the Chinese Academy of Sciences (Shanghai, China). The DU145 and PC3 human prostatic carcinoma cell lines were cultured in F-12K Nutrient Mixture (Gibco, USA), and LNCaP, 22RV1 were cultured in RPMI-1640 (Gibco, USA), WPMY-1 was cultured in DMEM (Gibco, USA), all supplemented with medium containing 10% fetal bovine serum (FBS, Gibco, USA) within a humidified atmosphere containing 5% CO2 at 37°C.

### Cell transfection

DU145 and PC3 were seeded in 6-well plates at 70% confluence the day before transfection. Cell transfection was performed with Lipofectamine 2000 (Invitrogen) according to the manufacturer's instructions. Six hours post-transfection, the culture medium was replaced with F-12K or RPMI-1640 containing fetal bovine serum. The sequences of the NR6A1 siRNA were: sense, 5′-GAGCAACCAUGGUGAUAGUTT-3′; and antisense, 5′-ACUAUCACCAUGGUUGCUCTT-3′. A random siRNA sequence was used as the negative control(NC): sense, 5′-UUCUCCGAACGUGUCACGUTT-3′; and antisense, 5′-ACGUGACACGUUCGGAGAATT -3′. For functional assays, cells grown in six-well plates were transfected with 100 pM of synthetic NR6A1 siRNA or NC. All siRNA or NC for each transfection were designed and synthesized by GenePharma (Shanghai, China).

### Plasmid constructs

The sequence of NR6A1 was synthesized and subcloned into pCDNA3.1 (GenePharma, Shanghai, China) vector. Ectopic expression of NR6A1 was achieved by using the pCDNA NR6A1 transfection and empty pCDNA vector (empty) was used as a control. The expression level of NR6A1 was detected by qPCR.

### RNA isolation and reverse transcription polymerase chain reaction (RT-PCR)

Total RNA was isolated from cultured cell lines using Trizol (Invitrogen) according to the manufacturer's instructions. Total RNA was transcribed into cDNAs using a PrimeScriptOne-Step RT-PCR Kit (TakaRa, Dalian, China) according to the manufacturer's instructions. RNA concentration and cDNAs concentration were measured using NanoDrop (Thermo Scientific). The following primer sequences of NR6A1 were used: forward primer,5′-GGGATGAACCGGAAGGCTATC-3′, and reverse primer, 5′-GGCTGGTTGCTCTCCGAAG-3′(synthesized by Invitrogen, Shanghai, China). The following PCR conditions for detecting cDNAs were used: 37°C for 15 minutes and 85°C for 5 seconds. The RT-PCR program was as follows: 95°C for 2 minutes,35 cycles of 95°C for 30 seconds, 60°C for 30 seconds, and 72°C for 1 minute. At last, 10ul PCR products were analyzed on 1.5% agarose (Gibco) gel electrophoresis with ethidium bromide by UV light transilluminator visualization. And the gray value was analyzed by gel image analysis BIO-RAD. The qRT-PCR program was as follows: 50°C for 2 minutes, 95°C for 5 minutes, 40 cycles of 95°C for 15 seconds, and 60°C for 60 seconds. The reactions were performed and analyzed using an Applied Biosystems StepOne Plus Real-Time PCR System (Applied Biosystems, USA). All reactions were run in triplicate.

### Cell cycle analysis

The cell cycle distribution was analyzed by flow cytometry (Becton Dickinson). The cells were cultured for 48h after transfection, then harvested, washed twice with ice-cold phosphate buffered saline and fixed with 70% ethanol for at least 12h at −20°C. The fixed cells were incubated in 50mg/ml of propidium iodide and 1 mg/ml of RNase for 30 minutes at room temperature. At least 20,000 cells were acquired for each sample. The experiments were performed in triplicate.

### Western blotting

Cells were washed 3 times in ice-cold phosphate buffered saline and lysed using radioimmunoprecipitation assay buffer (KeyGene Biotech) supplemented with protease inhibitors at 4°C for 30 min. Equal amounts of proteins were electrophoresed in 10% sodium dodecyl sulfate polyacrylamide gel electrophoresis, transferred onto a polyvinylidene fluoride (PVDF) membrane (Millipore, USA), blocked for 1 hour with 5% nonfat milk at room temperature, and incubated with primary antibodies at 4°C overnight. The membrane was incubated with a horseradish peroxidase-conjugated goat anti-rabbit secondary antibody at room temperature for 2 hours after three washes with Tris-buffered saline and 0.1% Tween. Antibodies against NR6A1(Abcam, UK), glyceraldehyde 3-phosphate dehydrogenase (GAPDH; Bioworld Technology, USA), E-cadherin, N-cadherin, and vimentin (Cell Signaling Technology, USA) were used in Western blot analysis in accordance with the manufacturer's instructions. The blots were detected using enhanced chemiluminescence (Thermo Scientific). Protein levels were determined by normalization to GAPDH.

### Cell migration and invasion assays

After siRNA transfection for 48 h, we used migration and invasion assays to examine whether NR6A1 was involved in the effect on metastasis on PCa cells. Each lower compartment of the Transwell (pore size, 8 mm; BD Bioscience) was filled with 500μL 0.5% FBS in medium to act as a chemoattractant. For the migration assays, 2 × 104 cells in 200 μL of serum-free medium were placed in the upper chamber of the Transwell. For the invasion assays, 5 × 104cells in 200μL of serum-free medium were placed in the upper chamber that was coated with Matrigel (BD Bioscience) in accordance with the manufacturer's protocol. After the cells had been incubated for 48 h at 37°C, the cells remaining in the upper membrane were removed completely by gentle swabbing. The number of cells that had invaded through the filter into the lower compartment was determined using a colorimetric crystal violet assay. With 200-time optical microscope, 5 scopes were randomly selected in each chamber to count. All of the experiments were performed in triplicate.

### Xenograft studies

The study was approved by Medical Laboratory Animal Welfare and Ethics Committee of Nanjing Medical University. BALB/c nude mice were randomly divided into three groups, and each contained four mice. Cells (5×10^6^ cells in 200μl) were suspended with 100μl PBS and 100μl Matrigel Matrix, and injected subcutaneously into the left armpit of each mouse. The volume and weight of the resulting tumors were measured every three days with calipers and electronic scale. The volume was calculated with the formula of length×width^2^×0.52. The mice were humanely sacrificed 3 weeks after injection, and the tumors were dissected. The methods were performed according to the approved guidelines.

### Statistical analyses

We used the Pearson correlation method to analyze the relationship between NR6A1 expression and clinicopathological factors. Univariate biochemical recurrence -free survival was assessed using the Kaplan–Meier curve and log-rank tests. Cox proportional hazards regression model was used to identify univariate and multivariate hazard ratios for the variables of this study. Results are expressed as mean ± standard deviation (SD). Differences *in vitro* between groups were subjected to Student's t-test. P < 0.05 was considered statistically significant. All of the statistical calculations were performed using SPSS software (Version 13.0 SPSS).

## SUPPLEMENTARY FIGURES


