# Supplementary material for: Positive expression of NR6A1/CT150 as a predictor of biochemical recurrence-free survival in prostate cancer patients
Source: Oncotarget. 2016 Aug 31;8(38):64427–39. doi: 10.18632/oncotarget.11749 (PMC5610014; doi:10.18632/oncotarget.11749)
Supplement: Supplementary file 1 [file oncotarget-08-64427-s001.pdf]

Positive expression of NR6A1/CT150 as a predictor of biochemical recurrence-free survival in prostate cancer patients

Supplementary Material

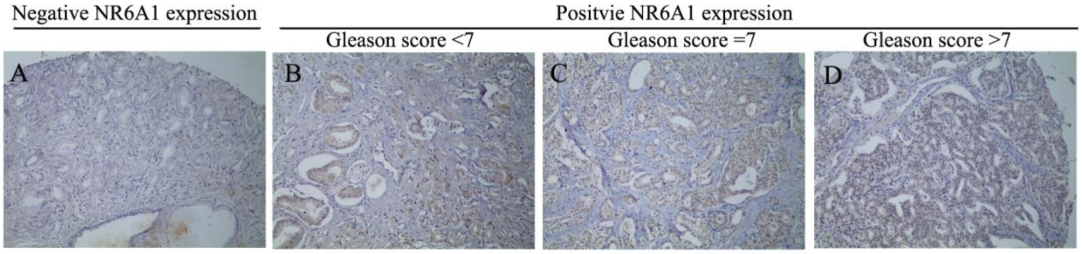

Fig.S1 NR6A1 protein expression in different Gleason score groups of prostate cancers. (A) Negative NR6A1 expression. (B-D) Positive NR6A1 expression in low/middle/high-grade prostate cacner.

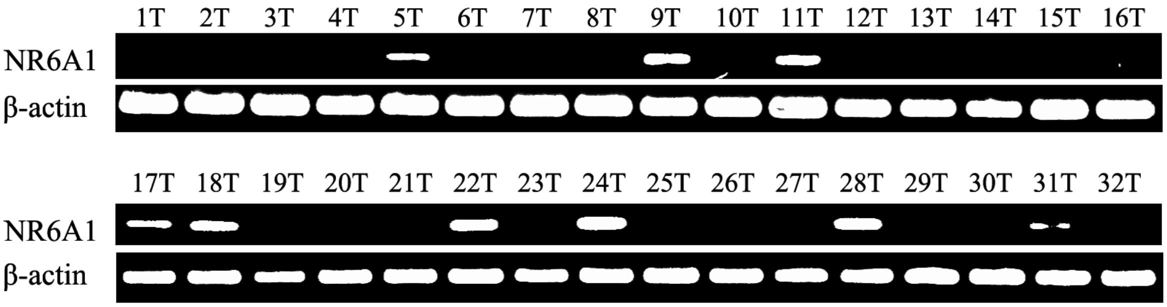

Fig.S2 Expression of NR6A1 in PCa tissues was evaluated by RT-PCR. β-actin was used as a positive control.

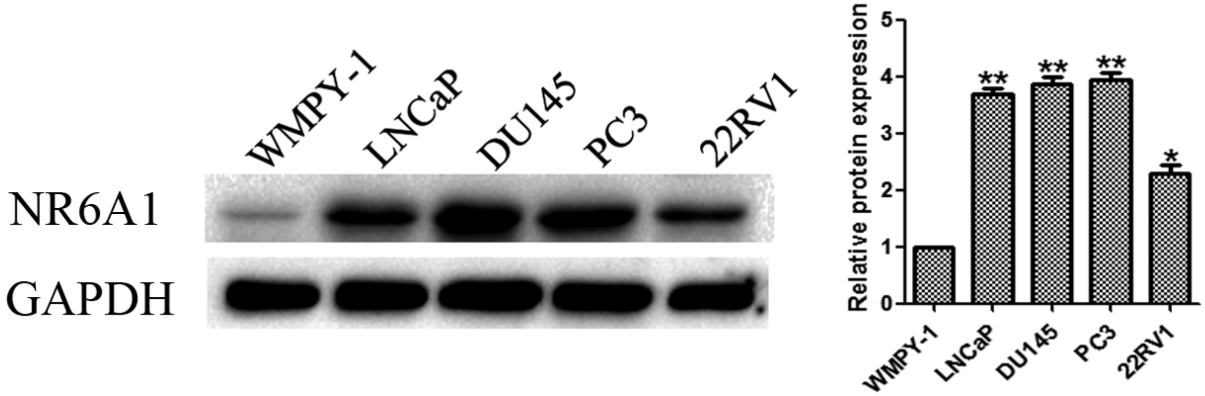

Fig.S3 NR6A1 protein expression in PCa cell lines (LNCaP, DU145, PC3 and 22RV1), normal prostate stromal cell (WPMY-1). GAPDH was used as a loading control. *$P < 0.05$ compared with Blank or NC; **$P < 0.01$ compared with Blank or NC.
